# Investigating the ADOS-2 calibrated severity score: insights from the ELENA cohort

**DOI:** 10.3389/frcha.2025.1674226

**Published:** 2026-01-12

**Authors:** Hugo Peyre, Amaria Baghdadli

**Affiliations:** 1 Centre de Ressource Autisme Languedoc-Roussillon et Centre d’Excellence sur l’Autisme et les Troubles du Neurodéveloppement (CeAND), CHU Montpellier, Montpellier, France; 2 UVSQ, Inserm, CESP, Team DevPsy, Université Paris-Saclay, Villejuif, France; 3 Faculté de Médecine, Université de Montpellier, Montpellier, France

**Keywords:** ADOS, autism, reliability, severity, validity

## Abstract

**Introduction:**

The Autism Diagnostic Observation Schedule (ADOS-2) is widely used to assess Autism Spectrum Disorder (ASD) symptom severity. To allow comparisons across modules and developmental levels, the Calibrated Severity Score (ADOS-CSS) was developed. However, concerns have been raised about potential changes in the measurement signal introduced by the calibration process, which may alter the signal captured by the ADOS-2 raw scores (ADOS-RS).

**Methods:**

Using longitudinal data from the ELENA cohort (*N* = 145 children with ASD assessed at baseline and after 3 years), we examined the psychometric properties of the ADOS-CSS and ADOS-RS. The Social Responsiveness Scale (SRS-2) was used as an external reference. Bootstrap resampling was used to estimate means and 95% confidence intervals (CIs).

**Results:**

Variance explained by module, age, and IQ was higher for the ADOS-RS (R^2^ = 0.45, 95% CI: 0.38–0.52) than for the ADOS-CSS (R^2^ = 0.16, 95% CI: 0.10–0.24). Stability of ASD severity scores over time was lower for the ADOS-CSS (*ρ* = 0.34, 95% CI: 0.23–0.45) than for the ADOS-RS (*ρ* = 0.60, 95% CI: 0.51–0.68). Correlations with SRS-2 scores were consistently lower for ADOS-CSS (*ρ* = 0.14, 95% CI: 0.06–0.22) than for ADOS-RS (*ρ* = 0.24, 95% CI: 0.17–0.32). Similar patterns were observed for change scores (Δ indicating score differences between baseline and follow-up): ΔSRS-2 correlated more strongly with ΔADOS-RS (*ρ* = 0.19, 95% CI: 0.09–0.28) than with ΔADOS-CSS (*ρ* = 0.13, 95% CI: 0.06–0.21). Differences between ADOS modules were reflected in SRS-2 and ADOS-RS, but markedly attenuated in ADOS-CSS, suggesting that calibration reduces module-related variance but also induces changes in the measurement signal that may affect sensitivity to individual differences.

**Discussion:**

While the ADOS-CSS facilitates standardized comparisons across modules, clinicians and researchers should be aware of potential distortions in the measurement signal associated with the calibration process.

**Clinical Trial Registration:**

NCT02625116.

## Introduction

The measurement of Autism Spectrum Disorder (ASD) symptoms severity is crucial for examining the developmental trajectories of social, communication and repetitive behavior functioning (1, 2). Both in clinical practice and research, the Autism Diagnostic Observation Schedule (ADOS-2) stands as a well-established instrument (3) for evaluating ASD symptoms in both the Social Affect (SA) and Restrictive and Repetitive Behaviors (RRB) domains. This instrument utilizes a range of standardized protocols depending on the age and language levels of the individuals being assessed. These protocols (including the ADOS-Toddler module and the modules 1–4) follow a similar structure, but the specific tasks and questions vary progressively across the different modules. Hence, symptom-based raw scores (i.e., ADOS Raw Scores – ADOS-RS) are not comparable across modules, due to variations in protocols and questions across modules. Therefore, the ADOS-2 Calibrated Severity Score (i.e., ADOS-CSS) was developed by Gotham et al. (4) to control for age, language level and modules. Since then, the ADOS-CSS has been widely adopted by clinicians and researchers (5) due to its relative independence from language level, modules, age, and verbal IQ (6–8), providing a distinct measure of ASD symptom severity and enabling tracking of symptom severity over time as participants change modules.

Although the ADOS-2 is widely considered the gold standard for clinical assessment, the calibration process used to compute the ADOS-CSS may introduce variability that, from a psychometric perspective, behaves like random or systematic measurement variability. Random measurement error refers to fluctuations in observed scores that are unrelated to true symptom severity, while systematic measurement bias refers to consistent over- or underestimation of symptom severity that shifts scores but does not necessarily reduce associations with other measures. Throughout this manuscript, we use the term “variability” rather than “error” to avoid implying suboptimal measurement, and to emphasize that such effects may arise from deliberate calibration choices rather than flaws in the instrument. This inquiry is based on several alternative hypotheses we propose regarding the interpretation of research on the ADOS-CSS to date. First, studies have shown that the ADOS-CSS is relatively independent of participant characteristics (such as language level, age and IQ) because they were found to account for a smaller variance explained [i.e., coefficient of determination (R^2^)] in regression models of the ADOS-CSS compared to the ADOS-RS (6–10). We propose an alternative interpretation suggesting that the reduction of R^2^ observed for the ADOS-CSS may reflect calibration-related changes in the measurement signal. Although the CSS transformation is deterministic, these changes in the measurement signal can psychometrically behave like added noise in downstream analyses. Such noise may potentially mask meaningful individual differences in symptom severity. Additionally, discordant results regarding changes in ASD severity have been reported when examining the ADOS-CSS and the ADOS-RS, with the former detecting less changes (11). Once more, we posit that this may be linked to the calibration process, which may introduce changes in the measurement signal that psychometrically behave like added noise. Second, several studies have reported a decrease in ADOS-RS scores with advanced ADOS-2 modules (e.g., from module 1 compared to module 3) (4, 12). However, this trend might reflect the progression of less severe cases into higher-level modules, and the calibration process, by adjusting for these differences, could inadvertently introduce systematic measurement bias.

To address these hypotheses, we investigated several psychometric properties of ADOS-2 scores (ADOS-CSS and ADOS-RS). The SRS-2 was used as an external comparative measure to examine the relative performance of ADOS-2 scores within predictive models (13). We evaluated reliability by examining the stability of scores over time (test–retest) and the consistency of scores across participants. Validity was assessed through multiple lenses: criterion validity via correlations with SRS-2 scores, construct validity via the proportion of variance in ADOS-2 scores explained by age, IQ, and module (R^2^) and regression coefficients reflecting expected patterns across modules, and sensitivity to change by examining whether ADOS-2 scores tracked changes in ASD symptoms over time. A lower R^2^ for the ADOS-CSS compared with the ADOS-RS indicates that calibration reduces the extent to which participant characteristics explain variance in observed scores, suggesting that calibration may introduce variability that diminishes construct validity, even if it standardizes scores across developmental levels. Similarly, the weaker correlations of ADOS-CSS with SRS-2 scores and the lower test–retest stability reflect potential impacts of calibration on criterion validity and reliability, while comparisons of changes over time indicate a possible reduction in sensitivity to detect symptom progression.

## Materials and methods

### Study sample

Participants in our study sample were recruited from the ELENA cohort (Longitudinal Study of Children with Autism), an ongoing prospective and multiregional cohort of children newly diagnosed ASD in specialized centers for neurodevelopmental disorders in France (14). In total, 876 children aged 2–16 years old were included in the ELENA cohort between 2013 and 2019. At inclusion (T0), and 3 years post-inclusion (T1), these children were assessed on various behavioral and cognitive characteristics. All children received a clinical diagnosis of ASD according to the DSM-5 criteria (15), confirmed by a multidisciplinary team using a standardized process, including the ADOS-2 (using the clinical thresholds of the ADOS-RS algorithm) and the ADI-Revised (16), administered by licensed and trained psychologists. Our study sample included a sub-sample of children from the ELENA cohort for whom both the ADOS-2 and the SRS-2 were available at inclusion and 3 years post-inclusion (*N* = 145). All participants included in the present study had complete data for total ADOS-CSS, ADOS-RS, and SRS-2 scores. Some item-level data were missing for the SA and RRB subdomains, as well as for IQ scores; no imputation was performed, and analyses for these variables were conducted with available data only.

At T0, children were assessed with the following ADOS-2 modules: toddler (*n* = 15), module 1 (*n* = 67), module 2 (*n* = 26), and module 3 (*n* = 37). At T1, modules were distributed as follows: module 1 (*n* = 74), module 2 (*n* = 31), and module 3 (*n* = 40) (see Supplementary Table S5). No participants were assessed with the toddler module at T1, reflecting developmental progression over the 3-year interval. Seventy-two children (50%) remained in the same module across time points, while 73 changed modules — most often transitioning from Toddler or Module 1 to more advanced modules.

### Assessment measures

#### ADOS-2

At T0 and T1, children were assessed with the module of the ADOS-2 appropriate to their age, language, and developmental level. The ADOS-CSS total score, ADOS-CSS Social Affect (SA) and the ADOS-CSS Restrictive and Repetitive Behaviors (RRB) span from 1 to 10, a higher score corresponding to greater severity of ASD symptoms.

For participants who changed ADOS-2 modules between T0 and T1, the ADOS-RS was calculated using items common across the relevant modules to ensure comparability over time. Participants who remained in the same module had their ADOS-RS calculated using the full item set. The questions change progressively between the modules (See Supplementary Tables S5, S6). As all the ADOS-RS scores were not based on the same number of questions for each child, we expressed the score as the average of the score per question. The ADOS-RS total, ADOS-RS SA and the ADOS-RS RRB scores were expressed with the same metric spanning from 0 to 2. Higher scores indicated more ASD symptoms.

#### SRS-2

Parents completed the French Social Responsiveness Scale, Second Edition [SRS-2 (17)]. The SRS-2 is a screening tool completed by parents that assesses ASD symptoms (18). Items are clustered into two DSM-5 diagnostic domains: Social Communication and Interaction (SCI), and Restricted Interest and Repetitive Behaviors (RIRB). For ease of reading, we refer to these domains as SRS-2 SA and SRS-2 RRB throughout the manuscript. The SRS-2 has demonstrated robust psychometric properties in the English version (17, 18) as well as in the French version (19). The SRS-2 total, SRS-2-SA and the SRS-2-RRB scores are converted in T-scores based on chronological age (normative population mean = 50, SD = 10). Higher scores indicated more ASD symptoms.

#### Intellectual functioning (IQ)

The Intellectual functioning or development (IQ – Intellectual Quotient) was calculated using standardized and validated instruments [i.e., Brunet-Lézine-R (20), BECS (21), PEP-3 (22), WPPSI-IV (23), WISC-V (24), WAIS-IV (25), K-ABC (26)] selected according to the child's age and developmental level following the approach used by Howlin et al. (27).

### Statistical analyses

#### Simulation of calibration-related variability

To assess whether calibration is associated with changes in the measurement signal (and, if so, to explore their potential magnitude), we generated nine new variables by adding varying levels of random noise to the original ADOS-RS scores. Specifically, we introduced a random component to the ADOS-RS in 10% increments. For instance, the variable ADOS-RS40 represents a composite score composed of 40% random noise and 60% of the original ADOS-RS score. To ensure the robustness of our findings, we repeated the analyses across 100 independently simulated datasets, each incorporating newly generated random noise. This procedure enabled us to systematically evaluate the influence of increasing noise levels on the measurement of autism symptom severity from a psychometric perspective.

We acknowledge that introducing random noise to mimic calibration is a heuristic approach and does not replicate how the ADOS-CSS transformation actually operates. The ADOS-CSS is derived through deterministic, empirically based mappings from raw totals to severity scores, reflecting normative and module-specific adjustments. Our rationale for using random noise is therefore conceptual: it provides a controlled method to evaluate the sensitivity of our analyses to added variability and to illustrate the potential impact of calibration-like effects on score distributions and associations, without implying that the procedure exactly reproduces the CSS algorithm. Consequently, this simulation framework does not allow inference about the calibration mechanism itself, but rather about the psychometric consequences of changes in the measurement signal.

#### Comparative analyses of ASD symptom severity scores

We compared ASD symptom severity scores (ADOS-CSS scores, ADOS-RS scores, and ADOS-RS10 to ADOS-RS90) in several ways. First, we employed linear regression models on our full sample (data from both T0 and T1), with ASD symptom severity score as the outcome variable and module, age, and IQ as independent variables. In these analyses, we focused on the R^2^ of each model. Second, we estimated the stability of ASD severity scores over time by examining the correlation between T0 and T1 measures. Third, we compared the correlation between ADOS scores and SRS-2 scores in our full sample. Fourth, we examined the correlation between changes over time (delta = T1 – T0) in ADOS-2 scores and changes in SRS-2 scores. This analysis aimed to compare the ability of the two ADOS-2 scores to track changes in autistic symptoms as measured by the SRS-2.

To assess whether calibration-related changes in the measurement signal were associated with differences in measurement validity, we examined the same linear regression models described previously, but here we focused on the parameter regression coefficient of the categorical variable corresponding to the module (module 3 was the reference group). We expect these standardized regression parameters to be lower for the ADOS-CSS than for the ADOS-RS. We interpreted closer alignment of SRS-2 regression parameters with those of the ADOS-RS (as opposed to the ADOS-CSS), as potential evidence of systematic changes in the measurement signal associated with calibration.

For all correlation analyses, we opted to use Spearman's correlation coefficient as it assesses the strength and direction of association between two ranked variables. This non-parametric method is advantageous as it does not require the data to be normally distributed.

To compare R^2^ values and correlation coefficients between ADOS-RS and ADOS-CSS, we performed a 1,000-iteration bootstrap. For each replicate, we recalculated the R^2^ and correlation coefficients and explicitly accounted for the dependence between paired measures (e.g., same participants measured on RS and CSS). Differences were assessed using empirical distributions: for R^2^, we computed ΔR^2^ = R^2^(ADOS-RS)−R^2^(ADOS-CSS) across replicates, and for correlation coefficients, we computed Δ*ρ* = *ρ*(ADOS-RS)−*ρ*(ADOS-CSS). The 95% confidence intervals of ΔR^2^ and Δ*ρ* were estimated from the percentiles of the bootstrap distributions, and *p*-values were obtained as the proportion of replicates in which ΔR^2^ or Δ*ρ* was ≤ 0. Differences between standardized regression coefficients across ADOS-RS, ADOS-CSS, and SRS-2 were assessed using *Z*-tests. Additional analyses were conducted separately for the SA and RRB domains. Data analyses were performed with SAS, version 9.3 (28). The significance level was defined as *p* < 0.05 for all tests.

## Results

### Sample characteristics

In our sample from the ELENA cohort (*N* = 145), the mean age at T0 was 5.41 years (SD = 2.66), and there were 20% of girls. Clinical information at T0 is provided in Table 1. For half of our sample the ADOS-2 module was the same between T0 and T1 (*N* = 72).

**Table 1:** Characteristics of the study sample (*N* = 145).

Follow-up (T1)	Specification	Baseline (T0)		Follow-up (T1)	
		% or mean	SD	% or mean	SD
Age (years)		5.4	2.7	8.5	2.7
Sex					
	Girls	20.0			
IQ		78.7	26.9		
ADOS-2 module					
	Module Toddler	10.3	2.7	0.0	
	Module 1	46.2		26.2	
	Module 2	17.9		26.2	
	Module 3	25.5		47.6	
SRS-2	Total score	96.2	19.2	83.7	16.2
ADOS-RS	Total score	1.1	0.4	1.1	0.4
ADOS-CSS	Total score	7.1	2.1	7.2	1.8
SRS-2	SA domain	92.3	17.9	80.5	15.3
ADOS-RS	SA domain	1.2	0.5	1.1	0.4
ADOS-CSS	SA domain	6.9	2.1	6.6	2.0
SRS-2	RRB domain	101.8	23.4	91.0	20.8
ADOS-RS	RRB domain	1.0	0.5	1.1	0.6
ADOS-CSS	RRB domain	7.3	2.1	8.1	1.8

ADOS-RS, ADOS raw scores; ADOS-CSS, ADOS calibrated severity score; SA, social affect; RRB, restrictive and repetitive behaviors (RRB); IQ, intellectual quotient.

### Variance explained, stability and correlation analyses

As expected, the variance explained by module, age, and IQ was much higher for the ADOS-RS [R^2^ = 0.45 (95% CI: 0.38–0.52)] than for the ADOS-CSS [R^2^ = 0.16 (95% CI: 0.10–0.24)] scores (*p*-value for the comparison of R^2^ < 0.001; Table 2). This proportion of variance explained reflects construct validity, showing that the ADOS-CSS captures less variability associated with participant characteristics than the ADOS-RS.

**Table 2:** Variance explained by module, age, and IQ in ASD severity scores.

Variables	R^2^ (IC 95%)^b^	*β*	*SD*	*p*-value
SRS-2	0.13 (0.08–0.19)			
ADOS-2 module^a^				
Module Toddler		1.01	0.32	<0.01
Module 1		0.92	0.21	<0.01
Module 2		0.30	0.19	0.12
IQ		0.00	0.00	0.87
Age		0.12	0.03	<0.01
ADOS-CSS	0.16 (0.10–0.24)			
ADOS-2 module^a^				
Module Toddler		0.29	0.32	0.38
Module 1		-0.30	0.21	0.16
Module 2		-0.41	0.19	0.04
IQ		-0.01	0.00	<0.01
Age		-0.07	0.03	0.02
ADOS-RS	0.45 (0.38–0.52)			
ADOS-2 module^a^				
Module Toddler		0.93	0.26	<0.01
Module 1		0.72	0.17	<0.01
Module 2		0.21	0.16	0.17
IQ		-0.01	0.00	<0.01
Age		-0.04	0.03	0.11

Standardized regression coefficients for ADOS-RS, ADOS-CSS, and SRS-2 are presented to illustrate the relative sensitivity of each measure to module, age, and IQ.

a Module 3 was the reference. ADOS-RS, ADOS raw scores; ADOS-CSS, ADOS calibrated severity score.

b Bootstrap resampling (1,000 iterations) was used to estimate mean values and their 95% confidence intervals.

The stability of ASD severity scores between T0 and T1 was moderate for the ADOS-RS, with a Spearman correlation coefficient of 0.60 [95% CI: 0.51–0.68], and lower for the ADOS-CSS, which showed a correlation of 0.34 [95% CI: 0.23–0.45] (*p*-value for the comparison of *ρ* < 0.001; Supplementary Table S2). These stability coefficients reflect reliability, indicating that the ADOS-CSS is less consistent over time than the ADOS-RS.

The SRS-2 showed a weak correlation with the ADOS-RS, with a coefficient of 0.24 [95% CI: 0.17–0.32], and an even lower correlation with the ADOS-CSS, at 0.14 [95% CI: 0.06–0.22] (*p*-value for the comparison of *ρ* < 0.001; Supplementary Table S3). These correlations provide evidence for criterion validity, showing that ADOS-RS scores align better with external measures of ASD symptoms than ADOS-CSS scores.

The change in SRS-2 (ΔSRS-2) was weakly correlated with the change in ADOS-RS, with a coefficient of 0.19 [95% CI: 0.09–0.28], and showed an even lower correlation with the change in ADOS-CSS, at 0.13 [95% CI: 0.06–0.21] (*p*-value for the comparison of *ρ* = 0.040; Supplementary Table S4). This analysis reflects sensitivity to change, indicating that ADOS-CSS may be less sensitive to detecting within-person changes over time.

When changes in the measurement signal comparable to adding 50% random noise were introduced to the ADOS-RS scores (ADOS-RS50), the patterns of variance explained, stability, and correlations closely resembled those of the ADOS-CSS. For example, R^2^ for total scores was 0.22 for ADOS-RS50 vs. 0.16 for ADOS-CSS (Supplementary Table S1), test-retest correlations were *ρ* = 0.31 vs. *ρ* = 0.34 (Supplementary Table S2), correlations with SRS-2 total scores were *ρ* = 0.17 vs. *ρ* = 0.14 (Supplementary Table S3) and correlations with change scores (delta T1–T0) were similar, with *ρ* = 0.12 for ADOS-RS50 compared with *ρ* = 0.13 for the ADOS-CSS (Supplementary Table S4).

### Regression coefficients and measurement validity

As anticipated, in our regression analysis (Table 2), the standardized regression coefficients of the categorical variable corresponding to the module were lower for the ADOS-CSS compared to the ADOS-RS. For instance, while the standardized regression coefficient associated with the toddler module (with module 3 as the reference) was 0.93 (SD = 0.32) for the ADOS-RS, it reduced to 0.29 (SD = 0.32) for the ADOS-CSS (*p*-value for the comparison = 0.157, not significant). Regression models using the ADOS-RS and SRS-2 yielded highly similar standardized coefficients across modules, suggesting a comparable sensitivity to module-related differences. Notably, the direction of the standardized regression coefficients was sometimes reversed in the ADOS-CSS compared to the ADOS-RS and SRS-2. For example, the standardized regression coefficient associated with module 2 was −0.41 (SD = 0.19), while it was 0.21 (SD = 0.16) and 0.30 (SD = 0.19) for the ADOS-RS (*p*-value for the comparison = 0.012) and SRS-2 (*p*-value for the comparison = 0.008), respectively (with module 3 as the reference). These coefficients reflect construct validity in that they show expected differences across modules; the reduction in ADOS-CSS coefficients suggests potential calibration-related suppression of meaningful module-related variation. While the SRS-2 exhibited independence from IQ, such independence was not observed for ADOS-2 scores. The SRS-2 was more correlated with the age of children than the ADOS-2 scores.

### Domain-specific analyses (SA and RRB)

The variance explained by module, age, and IQ was much higher for the ADOS-RS than for the ADOS-CSS scores in both the SA and RRB domains (*p*-values for the comparison of R^2^ < 0.001; Supplementary Table S1). Stability between T0 and T1 was lower for ADOS-CSS than for ADOS-RS in the SA domain (*p*-values for the comparison of *ρ* < 0.001) and RRB domain (*p*-values for the comparison <0.001; Supplementary Table S2). Correlations with SRS-2 were higher for ADOS-RS than ADOS-CSS in the SA domain (*p*-value for the comparison of *ρ* < 0.001), whereas correlations in the RRB domain were not significant for either score (Supplementary Table S3). This lack of significant correlation may reflect differences in the constructs assessed by the ADOS-RRB and SRS-2 RRB scales, and/or lower reliability of RRB measurement. Changes over time (ΔT1–T0) showed weak correlations with ΔSRS-2 scores for both ADOS-RS and ADOS-CSS, with no significant differences between the two measures in either domain (Supplementary Table S4).

## Discussion

### Impact of calibration on ASD symptom severity measurement

Accurately measuring ASD symptom severity is crucial for understanding developmental trajectories in social, communication, and repetitive behavior functioning, and the ADOS-2 is the gold standard for clinical assessment. In this study based on longitudinal data from the ELENA cohort, we investigated the potential impact of using the ADOS-2 Calibrated Severity Score (CSS) on the measurement signal of ASD symptoms. The results suggest that the calibration process alters the ASD symptom severity signal captured by the ADOS-2, inducing changes in the measurement signal comparable, in their psychometric consequences, to simulations in which approximately 50% random noise was added to the ADOS-RS. This sheds new light on the reduced coefficient of determination (R^2^) estimates in regression models of the ADOS-CSS compared to the ADOS-RS (6–10), which should not solely be interpreted as an indication of independence from certain characteristics (such as age, IQ or ADOS-2 modules). Rather, it suggests that calibration-related changes in the measurement signal may impact reliability-related indices of the ASD symptom signal. This reduction in R^2^ may also reflect properties of the CSS itself, including its restricted range (1–10) and potential ceiling or floor effects, which can compress variability and attenuate associations with external variables. These results extend concerns raised by previous study on the ADOS-2 module 4 (13) regarding the ADOS-CSS's ability to accurately capture the severity of social core symptoms of ASD.

### Concerns about longitudinal stability of ADOS-CSS scores

Our study questions certain conclusions regarding longitudinal ASD symptoms stability (7, 11). In our study, stability was operationalized as the correlation between instrument measures at T0 and T1. ADOS-CSS scores were found to be less stable over time than ADOS-RS scores, potentially resulting in a lower ability to track changes in ASD symptoms. This finding reflects a reduction in test–retest reliability, indicating that ADOS-CSS scores may be less consistent over time at the individual level compared with ADOS-RS scores. While previous work reported relative stability of CSS over 12–24 months in young children (7), those findings relied on group-level mean comparisons rather than individual-level stability indices. Thus, discrepancies between studies may reflect differences in the operational definition of longitudinal stability, which supports the interpretation that calibration may attenuate the sensitivity of CSS to detect within-person changes over time (7). This also highlights a potential reduction in sensitivity to change caused by calibration.

### Systematic changes in the measurement signal related to calibration

While differences in scores between modules were comparable between ADOS-RS and SRS-2, they were notably reduced for ADOS-CSS [which is one of the primary objectives of calibration (4, 12)]. This raises concerns about systematic changes in the measurement signal associated with calibration, particularly since the SRS-2, which is unaffected by ADOS module selection, preserved these differences. The calibration appears to suppress meaningful variability linked to the assessment context (i.e., the module administered). In this sense, the reduced sensitivity of the ADOS-CSS to module-based variation may reflect a loss of clinically relevant information, rather than an improvement in comparability. However, we were not able to quantify the exact magnitude of this bias, and our analyses addressed only one type of potential systematic change in the measurement signal related to calibration.

### Limitations and recommendations for future research

Given our moderate sample size (*N* = 145), these results should be interpreted with caution. Additionally, our study is limited by the relatively small number of participants within each module or module pair (when module changes occurred between the two evaluation time points) (see Supplementary Table S5). We encourage researchers with similar data to replicate our analyses to validate or challenge our results. Finally, our study examined only one type of potential systematic change in the measurement signal related to calibration, and other sources of calibration-related effects remain to be explored.

## Conclusion

These findings highlight that while ADOS-CSS provides standardized scores across modules, calibration-related changes in the measurement signal may reduce the sensitivity of the instrument to meaningful individual differences in ASD symptom severity, suggesting caution when interpreting severity estimates in longitudinal studies or clinical assessments. Our study offers crucial insights into the implications of calibration on measuring ASD symptom severity in clinical practice and research.

## Data Availability

The data analyzed in this study is subject to the following licenses/restrictions: The datasets used and analysed during the current study are available from the corresponding author upon reasonable request. Requests to access these datasets should be directed to hugo.peyre@chu-montpellier.fr.
