# Supplementary material for: Investigating the ADOS-2 calibrated severity score: insights from the ELENA cohort
Source: Front Child Adolesc Psychiatry. 2026 Jan 12;4:1674226. doi: 10.3389/frcha.2025.1674226 (PMC12833228; doi:10.3389/frcha.2025.1674226)
Supplement: Supplementary file 1 [file Supplementaryfile1.docx]

**Supplementary Table 1.** Variance explained by module, age, and IQ in ASD severity scores. Standardized regression coefficients for ADOS-RS, ADOS-CSS, and SRS-2 are presented to illustrate the relative sensitivity of each measure to module, age, and IQ.

|  | Total | | | |  | SA | | | |  | RRB | | | |
| --- | --- | --- | --- | --- | --- | --- | --- | --- | --- | --- | --- | --- | --- | --- |
|  | R² (CI-95%)^#^ | β | *SD* | p-value |  | R² (CI-95%)^#^ | β | *SD* | p-value |  | R² (CI-95%)^#^ | β | *SD* | p-value |
| **SRS-2** | 0.13 (0.08-0.19) |  |  |  |  | 0.10 (0.05-0.18) |  |  |  |  | 0.10 (0.05-0.17) |  |  |  |
| ADOS-2 module* |  |  |  |  |  |  |  |  |  |  |  |  |  |  |
| Module Toddler |  | 1.01 | 0.32 | <0.01 |  |  | 1.11 | 0.32 | <0.01 |  |  | 0.44 | 0.33 | 0.19 |
| Module 1 |  | 0.92 | 0.21 | <0.01 |  |  | 0.90 | 0.21 | <0.01 |  |  | 0.84 | 0.22 | <0.01 |
| Module 2 |  | 0.30 | 0.19 | 0.12 |  |  | 0.29 | 0.19 | 0.13 |  |  | 0.33 | 0.20 | 0.10 |
| IQ |  | 0.00 | 0.00 | 0.87 |  |  | 0.00 | 0.00 | 0.81 |  |  | 0.00 | 0.00 | 0.97 |
| Age |  | 0.12 | 0.03 | <0.01 |  |  | 0.11 | 0.03 | <0.01 |  |  | 0.11 | 0.03 | <0.01 |
| **ADOS-CSS** | 0.16 (0.10-0.24) |  |  |  |  | 0.12 (0.08-0.18) |  |  |  |  | 0.14 (0.09-0.21) |  |  |  |
| ADOS-2 module* |  |  |  |  |  |  |  |  |  |  |  |  |  |  |
| Module Toddler |  | 0.29 | 0.32 | 0.38 |  |  | 0.36 | 0.34 | 0.28 |  |  | -0.98 | 0.33 | <0.01 |
| Module 1 |  | -0.30 | 0.21 | 0.16 |  |  | -0.37 | 0.22 | 0.09 |  |  | -0.18 | 0.21 | 0.40 |
| Module 2 |  | -0.41 | 0.19 | 0.04 |  |  | -0.47 | 0.20 | 0.02 |  |  | -0.22 | 0.19 | 0.26 |
| IQ |  | -0.01 | 0.00 | <0.01 |  |  | -0.01 | 0.00 | <0.01 |  |  | 0.00 | 0.00 | 0.07 |
| Age |  | -0.07 | 0.03 | 0.02 |  |  | -0.01 | 0.03 | 0.67 |  |  | -0.14 | 0.03 | <0.01 |
| **ADOS-RS** | 0.45 (0.38-0.52) |  |  |  |  | 0.39 (0.32-0.45) |  |  |  |  | 0.32 (0.17-0.30) |  |  |  |
| ADOS-2 module* |  |  |  |  |  |  |  |  |  |  |  |  |  |  |
| Module Toddler |  | 0.93 | 0.26 | <0.01 |  |  | 1.37 | 0.28 | <0.01 |  |  | -0.45 | 0.29 | 0.12 |
| Module 1 |  | 0.72 | 0.17 | <0.01 |  |  | 0.67 | 0.18 | <0.01 |  |  | 0.45 | 0.19 | 0.02 |
| Module 2 |  | 0.21 | 0.16 | 0.17 |  |  | 0.05 | 0.17 | 0.75 |  |  | 0.40 | 0.17 | 0.02 |
| IQ |  | -0.01 | 0.00 | <0.01 |  |  | -0.01 | 0.00 | <0.01 |  |  | -0.01 | 0.00 | 0.01 |
| Age |  | -0.04 | 0.03 | 0.11 |  |  | 0.00 | 0.03 | 0.98 |  |  | -0.11 | 0.03 | <0.01 |
| **ADOS-RS40** | 0.30 |  |  |  |  | 0.27 |  |  |  |  | 0.22 |  |  |  |
| ADOS-2 module* |  |  |  |  |  |  |  |  |  |  |  |  |  |  |
| Module Toddler |  | 0.56 | - | - |  |  | 0.83 | - | - |  |  | -0.27 | - | - |
| Module 1 |  | 0.43 | - | - |  |  | 0.40 | - | - |  |  | 0.27 | - | - |
| Module 2 |  | 0.13 | - | - |  |  | 0.03 | - | - |  |  | 0.24 | - | - |
| IQ |  | -0.01 | - | - |  |  | -0.01 | - | - |  |  | 0.00 | - | - |
| Age |  | -0.02 | - | - |  |  | 0.00 | - | - |  |  | -0.07 | - | - |
| **ADOS-RS50** | 0.22 |  |  |  |  | 0.19 |  |  |  |  | 0.16 |  |  |  |
| ADOS-2 module* |  |  |  |  |  |  |  |  |  |  |  |  |  |  |
| Module Toddler |  | 0.46 | - | - |  |  | 0.68 | - | - |  |  | -0.23 | - | - |
| Module 1 |  | 0.36 | - | - |  |  | 0.33 | - | - |  |  | 0.21 | - | - |
| Module 2 |  | 0.10 | - | - |  |  | 0.02 | - | - |  |  | 0.19 | - | - |
| IQ |  | -0.01 | - | - |  |  | -0.01 | - | - |  |  | 0.00 | - | - |
| Age |  | -0.02 | - | - |  |  | 0.00 | - | - |  |  | -0.06 | - | - |
| **ADOS-RS60** | 0.13 |  |  |  |  | 0.12 |  |  |  |  | 0.10 |  |  |  |
| ADOS-2 module* |  |  |  |  |  |  |  |  |  |  |  |  |  |  |
| Module Toddler |  | 0.38 | - | - |  |  | 0.56 | - | - |  |  | -0.19 | - | - |
| Module 1 |  | 0.29 | - | - |  |  | 0.27 | - | - |  |  | 0.17 | - | - |
| Module 2 |  | 0.09 | - | - |  |  | 0.02 | - | - |  |  | 0.15 | - | - |
| IQ |  | 0.00 | - | - |  |  | 0.00 | - | - |  |  | 0.00 | - | - |
| Age |  | -0.02 | - | - |  |  | 0.00 | - | - |  |  | -0.04 | - | - |
| * Module 3 was the reference. ADOS-RS: ADOS Raw Scores; ADOS-CSS: ADOS Calibrated Severity Score; SA: Social Affect; RRB: Restrictive and Repetitive Behaviors (RRB); IQ; Intellectual Quotient. ADOS-RS40 represented a combination of 40% of a random variable + 60% of the ADOS-RS score. ^#^ Bootstrap resampling (1000 iterations) was used to estimate mean values and their 95% confidence intervals. | | | | | | | | | | | | | | |

**Supplementary Table 2.** Stability of ASD severity scores: Spearman correlation estimates between T0 and T1 for SRS-2, ADOS-CSS, and ADOS-RS.

|  | Total | |  | SA | |  | RRB | |
| --- | --- | --- | --- | --- | --- | --- | --- | --- |
|  | Correlation (T0 with T1) | |  | Correlation (T0 with T1) | |  | Correlation (T0 with T1) | |
|  | *ρ* (CI-95%)^#^ | p-value |  | *ρ* (CI-95%)^#^ | p-value |  | *ρ* (CI-95%)^#^ | p-value |
| SRS-2 | 0.47 (0.39-0.56) | 0.00 |  | 0.52 (0.41-0.61) | 0.00 |  | 0.45 (0.34-0.56) | 0.00 |
| ADOS-CSS | 0.34 (0.23-0.45) | 0.00 |  | 0.28 (0.16-0.40) | 0.00 |  | 0.20 (0.06-0.32) | 0.01 |
| ADOS-RS | 0.60 (0.51-0.68) | 0.00 |  | 0.48 (0.37-0.59) | 0.00 |  | 0.36 (0.24-0.47) | 0.00 |
| ADOS-RS40 | 0.43 | - |  | 0.35 | - |  | 0.26 | - |
| ADOS-RS50 | 0.31 | - |  | 0.25 | - |  | 0.18 | - |
| ADOS-RS60 | 0.19 | - |  | 0.16 | - |  | 0.11 | - |
| *ρ*: Spearman’s correlation coefficient. ADOS-RS: ADOS Raw Scores; ADOS-CSS: ADOS Calibrated Severity Score; SA: Social Affect; RRB: Restrictive and Repetitive Behaviors (RRB). ADOS-RS40 represented a combination of 40% of a random variable + 60% of the ADOS-RS score. ^#^ Bootstrap resampling (1000 iterations) was used to estimate mean values and their 95% confidence intervals. | | | | | | | | |

**Supplementary Table 3.** Correlation of ADOS-2 Scores with SRS-2.

|  |  |  |  |  |  |  |  |  |
| --- | --- | --- | --- | --- | --- | --- | --- | --- |
|  | Correlation | |  | Correlation | |  | Correlation | |
|  | with SRS-2 Total Score | |  | with SRS-2 SA | |  | with SRS-2 RRB | |
|  | *ρ* (CI-95%)*^#^* | p-value |  | *ρ* (CI-95%) | p-value |  | *ρ* (CI-95%) | p-value |
| ADOS-CSS | 0.14 (0.06-0.22) | 0.02 |  | 0.16 (0.08-0.25) | 0.00 |  | -0.02 (-0.11-0.08) | 0.99 |
| ADOS-RS | 0.24 (0.17-0.32) | 0.00 |  | 0.22 (0.14-0.32) | 0.00 |  | 0.04 (-0.04-0.14) | 0.46 |
| ADOS-RS40 | 0.20 | - |  | 0.23 | - |  | 0.03 | - |
| ADOS-RS50 | 0.17 | - |  | 0.19 | - |  | 0.03 | - |
| ADOS-RS60 | 0.13 | - |  | 0.15 | - |  | 0.02 | - |
| *ρ*: Spearman’s correlation coefficient. ADOS-RS: ADOS Raw Scores; ADOS-CSS: ADOS Calibrated Severity Score; SA: Social Affect; RRB: Restrictive and Repetitive Behaviors (RRB). ADOS-RS40 represented a combination of 40% of a random variable + 60% of the ADOS-RS score. ^#^ Bootstrap resampling (1000 iterations) was used to estimate mean values and their 95% confidence intervals. | | | | | | | | |

**Supplementary Table 4.** Correlation of delta (T1-T0) SRS-2 scores with delta (T1-T0) ADOS-2 scores.

|  | Correlation | |  | Correlation | |  | Correlation | |
| --- | --- | --- | --- | --- | --- | --- | --- | --- |
|  | with delta (T1-T0) SRS-2 Total Score | |  | with delta (T1-T0) SRS-2 SA | |  | with delta (T1-T0) SRS-2 RRB | |
|  | *ρ* (CI-95%)*^#^* | p-value |  | *ρ* (CI-95%)*^#^* | p-value |  | *ρ* (CI-95%)*^#^* | p-value |
| Delta (T1-T0) ADOS-CSS | 0.13 (0.06-0.21) | 0.11 |  | 0.18 (0.08-0.27) | 0.11 |  | 0.03 (-0.08-0.12) | 0.72 |
| Delta (T1-T0) ADOS-RS | 0.19 (0.09-0.28) | 0.02 |  | 0.18 (0.07-0.29) | 0.03 |  | 0.00 (-0.11-0.10) | 0.97 |
| Delta (T1-T0) ADOS-RS40 | 0.15 | - |  | 0.15 | - |  | 0.00 | - |
| Delta (T1-T0) ADOS-RS50 | 0.12 | - |  | 0.12 | - |  | 0.00 | - |
| Delta (T1-T0) ADOS-RS60 | 0.09 | - |  | 0.09 | - |  | 0.01 | - |
| *ρ*: Spearman’s correlation coefficient. ADOS-RS: ADOS Raw Scores; ADOS-CSS: ADOS Calibrated Severity Score; SA: Social Affect; RRB: Restrictive and Repetitive Behaviors (RRB); ADOS-RS40 represented a combination of 40% of a random variable + 60% of the ADOS-RS score. ^#^ Bootstrap resampling (1000 iterations) was used to estimate mean values and their 95% confidence intervals. | | | | | | | | |

**Supplementary Table 5.** Description of modules administered at both time points, participant counts, and number of ADOS-2 items.

| ADOS2 module at T0 | ADOS2 module at T1 | N | Number of items in the SA domain | Number of items in the RRB domain | Total number of items |
| --- | --- | --- | --- | --- | --- |
| Toddler | 1 | 7 | 10 | 3 | 13 |
|  | 2 | 6 | 8 | 3 | 11 |
|  | 3 | 2 | 7 | 3 | 10 |
| **1** | **1** | **30** | **11** | **5** | **16** |
|  | 2 | 26 | 8 | 5 | 13 |
|  | 3 | 11 | 6 | 4 | 10 |
| **2** | 1 | 1 | 8 | 4 | 12 |
|  | **2** | **6** | **10** | **4** | **14** |
|  | 3 | 19 | 8 | 4 | 12 |
| **3** | **3** | **37** | **10** | **4** | **14** |
| When participants had the same ADOS-2 module at both time points, it is indicated in bold. | | | | | |

**Supplementary Table 6a.** ADOS-Toddler: Common Symptoms with Module 1, Module 2, and Module 3.

| **ADOS-Toddler** | **Common Symptoms with Module 1** | **Common Symptoms with Module 2** | **Common Symptoms with Module 3** |
| --- | --- | --- | --- |
| **COMA2** |  |  |  |
| **COMA7** | SSA | SSA | SSA |
| **COMA8** | SSA | SSA | SSA |
| **ISRB1** | SSA | SSA | SSA |
| **ISRB4** | SSA | SSA | SSA |
| **ISRB5** | SSA |  |  |
| **ISRB6** | SSA | SSA | SSA |
| **ISRB12** | SSA | SSA | SSA |
| **ISRB13** | SSA | SSA |  |
| **ISRB14** | SSA |  |  |
| **ISRB15** | SSA | SSA | SSA |
| **CSIRA3** | RRB |  |  |
| **CSIRD1** | RRB | RRB | RRB |
| **CSIRD2** | RRB | RRB | RRB |
| **CSIRD5** |  | RRB | RRB |

**Supplementary Table 6b.** ADOS-Module 1: Common Symptoms with Module 2 and Module 3.

| **ADOS-Module 1** | **Common Symptoms with Module 2** | **Common Symptoms with Module 3** |
| --- | --- | --- |
| **COMA2** |  |  |
| **COMA7** | SSA | SSA |
| **COMA8** | SSA | SSA |
| **ISRB1** | SSA | SSA |
| **ISRB3** | SSA | SSA |
| **ISRB4** |  |  |
| **ISRB5** | SSA | SSA |
| **ISRB9** | SSA |  |
| **ISR10** | SSA |  |
| **ISRB11** |  |  |
| **ISRB12** | SSA | SSA |
| **CSIRA3** | RRB |  |
| **CSIRA5** | RRB | RRB |
| **CSIRD1** | RRB | RRB |
| **CSIRD2** | RRB | RRB |
| **CSIRD4** | RRB | RRB |

**Supplementary Table 6c.** ADOS-Module 2: Common Symptoms with Module 1 and Module 3.

| **ADOS-Module 2** | **Common Symptoms with Module 3** | **Common Symptoms with Module 1** |
| --- | --- | --- |
| **COMA6** |  | SSA |
| **COMA7** | SSA | SSA |
| **ISRB1** | SSA | SSA |
| **ISRB2** | SSA | SSA |
| **ISRB3** | SSA | SSA |
| **ISRB5** | SSA | SSA |
| **ISRB6** |  | SSA |
| **ISRB8** | SSA | SSA |
| **ISRB11** | SSA |  |
| **ISRB12** | SSA |  |
| **CSIRA4** | RRB | RRB |
| **CSIRD1** | RRB | RRB |
| **CSIRD2** | RRB | RRB |
| **CSIRD4** | RRB | RRB |
